# Newborn Screening of Primary Carnitine Deficiency: An Overview of Worldwide Practices and Pitfalls to Define an Algorithm before Expansion of Newborn Screening in France

**DOI:** 10.3390/ijns9010006

**Published:** 2023-02-01

**Authors:** Charles R. Lefèvre, François Labarthe, Diane Dufour, Caroline Moreau, Marie Faoucher, Paul Rollier, Jean-Baptiste Arnoux, Marine Tardieu, Léna Damaj, Claude Bendavid, Anne-Frédérique Dessein, Cécile Acquaviva-Bourdain, David Cheillan

**Affiliations:** 1 Rennes University Hospital Center, 35033 Rennes, France; 2 Reference Center of Inherited Metabolic Disorders, Clocheville Hospital, 37000 Tours, France; 3 Reference Center for Inborn Error of Metabolism, Department of Pediatrics, Necker-Enfants Malades Hospital, APHP, 75015 Paris, France; 4 Metabolism and Rare Disease Unit, Department of Biochemistry and Molecular Biology, Center of Biology and Pathology, Lille University Hospital Center, 59000 Lille, France; 5 Center for Inherited Metabolic Disorders and Neonatal Screening, East Biology and Pathology Department, Groupement Hospitalier Est (GHE), Hospices Civils de Lyon, 69500 Bron, France

**Keywords:** primary carnitine deficiency, CDSP, PCD, CTD, CUD, newborn screening, NBS

## Abstract

Primary Carnitine Deficiency (PCD) is a fatty acid oxidation disorder that will be included in the expansion of the French newborn screening (NBS) program at the beginning of 2023. This disease is of high complexity to screen, due to its pathophysiology and wide clinical spectrum. To date, few countries screen newborns for PCD and struggle with high false positive rates. Some have even removed PCD from their screening programs. To understand the risks and pitfalls of implementing PCD to the newborn screening program, we reviewed and analyzed the literature to identify hurdles and benefits from the experiences of countries already screening this inborn error of metabolism. In this study, we therefore, present the main pitfalls encountered and a worldwide overview of current practices in PCD newborn screening. In addition, we address the optimized screening algorithm that has been determined in France for the implementation of this new condition.

## 1. Introduction

Primary Carnitine Deficiency (PCD) (OMIM #212140)—also referred to as systemic primary carnitine deficiency (CDSP), carnitine transporter defect (CTD), or carnitine uptake deficiency (CUD)—is an autosomal recessive inborn error of metabolism involving a disorder of the carnitine cycle. It is a part of fatty acid oxidation (FAO) disorders and is caused by a partial or complete loss of function of the membrane transporter organic cation/carnitine transporter novel 2 (OCTN2). This solute carrier is coded by the *SLC22A5* gene, comprising 10 exons, located approximately on a 26 kb region on chromosome 5q31.1 (chr5:132,369,710–132,395,612) [1,2]. This sodium-dependent carnitine symporter is the main carnitine (3-hydroxy-4-(trimethylazaniumyl)butanoate) transporter in mammals, displaying a high affinity for carnitine (K_M_ = 4.3 µmol·L^−1^) [3]. OCTN2 is ubiquitous, with expression predominantly in kidney and intestinal cells, to ensure absorption and reabsorption of L-carnitine, and in skeletal muscles, to allow the shuttling of long chain fatty acids across inner mitochondrial membranes toward the fatty acid oxidation process [4]. Carnitine is almost exclusively intracellular (>99% of the total pool), with high tissue concentrations [5]. Carnitine homeostasis is balanced by dietary intake, endogenous biosynthesis, and especially by renal reabsorption. There are compensatory mechanisms, thus even a poor carnitine diet or a defect in carnitine biosynthesis does not affect FAO [6,7]. However, when OCTN2 function is impaired, a major urinary leak of free carnitine leads to a progressively significant decrease in both intracellular and circulating carnitine concentrations, resulting in PCD. Clinical characteristics of PCD encompass a broad clinical spectrum and have been widely assessed in high quality reviews [8,9,10,11]. In absence of newborn screening (NBS), patients usually present in their infancy: acute metabolic decompensation with hypoketotic hypoglycemia; dilated cardiomyopathy; and hepatic cytolysis. Without L-carnitine treatment, death can occur due to heart failure. Fortunately, PCD has an excellent prognosis upon L-carnitine supplementation and almost all patients remain asymptomatic [11]. Incidence of primary carnitine deficiency was quite variable depending on the studied population, ranging from 1:300 in the Faroe Islands [12] where there was a founding mutation, to 1:30-142,000 in Japan, Australia, or USA [13,14,15]. Regarding the incidence, the knowledge of this disease’s natural history, and the availability of a safe and efficient treatment, PCD follows consolidated principles for newborn screening [16], especially as free carnitine (C_0_) represents an easily measurable biomarker on dried blood spot (DBS) [17]. New South Wales (Australia) was the first state to evaluate PCD newborn screening in the late 1990s [18], and this was usually conducted by expanded newborn screening programs deployed since then [19,20,21]. Nevertheless, screening of primary carnitine deficiency is not simple, due to various secondary carnitine deficiencies that may generate false-positives (e.g., maternal carnitine deficiency, organic acidurias, pivalic acid-based antibiotherapy, pre-term birth, etc.) which represent pitfalls for the diagnosis and management of newborn PCD. Consequently, several algorithms for screening have been proposed, which include: different thresholds for C_0_ and other biomarkers; molecular sequencing of *SLC22A5*; and functional confirmation by carnitine uptake assay on skin fibroblasts. In this study, we aimed to review the situation of PCD newborn screening worldwide before the expansion of newborn screening in France at the beginning of 2023 [22], by gathering epidemiological, biological, and molecular data, to set an appropriate screening algorithm.

## 2. Worldwide Overview of Primary Carnitine Deficiency Newborn Screening

### 2.1. Countries/Regions Screening PCD

**Figure 1:**
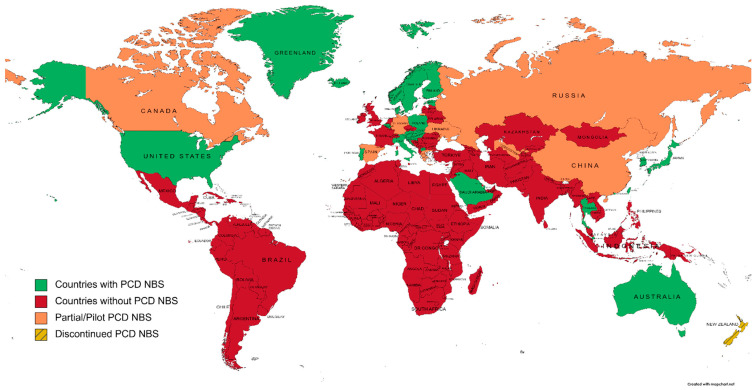
A world map of primary carnitine deficiency newborn screening programs, according to national NBS societies and the literature (created on mapchart.net, accessed on 27 October 2022).

To evaluate the extent of PCD NBS worldwide, we have screened national NBS programs and the literature for countries/regions that have implemented this condition. Actual NBS programs including PCD and excluding PCD are represented in Figure 1.

#### 2.1.1. Australia and New Zealand

Australia was the first country to include PCD in NBS [18], in 1998. The cut-off for low free carnitine to trigger a retest during screening was set to 10 µmol·L^−1^. A confirmed level of C_0_ < 5 µmol·L^−1^ generated a second sample, and PCD diagnosis was confirmed through OCTN2 activity on fibroblasts. To date, PCD NBS is performed nationwide [23].

New Zealand implemented PCD to ENBS in 2006, with a screen-positive level of C_0_ of 5 µmol·L^−1^ and molecular confirmation [24]. However, due to the low incidence (two cases in ten years; 1:300,000 births); the prevalence of asymptomatic patients; and the impact of diagnosing more mothers with PCD than newborns, PCD screening was considered unsuitable for NBS and was therefore discontinued [25].

#### 2.1.2. North America

North America has almost a full coverage of PCD screening. Newborn screening has been nationally organized since mid-1980s under the aegis of the Council of Regional Networks for Genetic Services (CORN) [26]. In 2006, the American College of Medical Genetics (ACMG) provided guidelines to promote a standardized and uniform newborn screening program [27]. These guidelines had a substantial impact on perinatal healthcare through early identification and treatment of inborn errors of metabolism, including PCD, reducing morbidity and mortality [20,28,29]. Consequently, the USA has a solid nationwide background on NBS, its follow-up and outcomes [30], enhanced by standardization tools and programs [31].

English-speaking Canadian regions follow the ACMG’s guidelines [32,33,34], whereas French-Canadian provinces do not screen DBS for PCD even though they provide an interesting urinary NBS for organic acidurias, urea cycle disorders, cystinuria, homocystinuria, and creatine synthesis and transport disorders [34].

#### 2.1.3. Central and South America

Mexico and South America started NBS in mid-1970s with PKU and struggled to achieve a whole coverage of population since then, with the exception of Cuba, Costa Rica, Chile, and Uruguay, where around 100% coverage was reached [35,36]. Difficulties encountered were mostly due to the lack of financial resources and fundings for free national NBS programs (especially regarding the high cost of tandem MS equipment), and the high prevalence of major, priority health issues, such as malnutrition and infectious diseases. As a result, most Central and South American countries have NBS programs, but to date, none of them include PCD, and only Costa Rica and Uruguay have set up a tandem MS NBS program [37]. It is to be noted that French Guyana and French Polynesia follow French guidelines for NBS.

#### 2.1.4. Europe

PCD NBS in Europe is heterogeneous. Germany was the first country to include PCD to their program in a pilot study between 1998 and 2001 [38]. Biomarkers used for the screening were C_0_ < 10 µmol·L^−1^ and total acylcarnitines (C_3_ to C_18_) of <10 µmol·L^−1^. Confirmation was biochemically deduced by determining OCTN2 activity on fibroblasts. In 2007, only Austria, Belgium, Denmark, and Poland had PCD on their ENBS panel [19]. NBS for Greenland and Faroe Islands are managed by Denmark. In Faroese population, there was a high incidence (~1:300) of PCD caused by a founder pathogenic variant on *SLC22A5*: c.95A>G, p.(Asn32Ser) [39]. Consequently, to minimize false negatives on NBS and to ensure diagnosis of all PCD patients, a nationwide second screening performed at two months of age had been introduced [40]. In addition, all Scandinavian countries have included PCD to NBS, and few other countries have conducted pilot studies or have already began to screen PCD, such as Portugal or Italy [21,41]. It is to be noted that the UK does not screen PCD, to date, even though in 2009, England had conducted a pilot study of amino acid and acylcarnitines analysis on cord blood samples to identify inborn errors of metabolism [42]. The Netherlands have listed PCD, along with seven other diseases, in the upcoming schedule of NBS expansion [43], and France will be including PCD to NBS at the beginning of 2023.

#### 2.1.5. Africa

Middle East and North Africa (MENA) is a large region consisting of 21 countries; from Morocco in northwestern Africa, to Iran in southwestern Asia. Genetic disorders are relatively common in this area due to the high rate of consanguinity [44]. Efforts have allowed NBS programs to emerge, resulting from pilot programs and successful studies [45,46,47]. However, to date, only Qatar and Saudi Arabia have included PCD in their programs [48,49,50].

Regarding Sub-Saharan Africa, NBS implementation is still at its beginning. However, much efforts are being made by a Pan-African Workshop on Newborn Screening [51]. Sickle Cell Disease (SCD) is the disease with the highest prevalence in this region and is, therefore, of priority and collaborations will be needed to expand NBS toward a larger panel in the future.

#### 2.1.6. Asia

Teams in Asia were the first to elude that pathogenic variations in the *SLC22A5* gene was the molecular basis of primary carnitine deficiency [14,52,53]. Incidence of PCD appears to be more frequent in Asian populations than in those from western countries, even being one of the most prevalent inherited metabolic diseases in the Chinese population [54]. However, the first pilot study of ESI-MS/MS-based NBS in Japan, led by Schigematsu et al., did not report any cases of PCD. First Asian studies and experience on PCD NBS started in late 2000s in China (province-based program), Taiwan, South Korea, and Japan. More recently, Thailand and Philippines included PCD to the ENBS program as well [41,55,56]. To our knowledge, India still struggles to initiate a nationwide NBS program, and other Asian Pacific or Central Asia countries have not included PCD to their program to date [57]. The high incidence of PCD in Asian populations, along with the development of Next Generation Sequencing, have led to the emergence of a systematic study of the *SLC22A5* gene as a second-tier testing after phenotypic screening [58,59,60].

#### 2.1.7. Russia

As it is part of both Europe and Asia, Russia is addressed as a separate entity. To date, the nationwide NBS program in Russia includes: phenylketonuria, congenital hypothyroidism, congenital adrenal hyperplasia, galactosemia, and cystic fibrosis. However, Primorsky and Moscow regions are currently performing tandem MS ENBS to identify 39 and 11 diseases, respectively, and the national expansion of NBS is being discussed [61].

## 3. Reports of NBS for PCD Worldwide

We screened the literature for regional or nationwide studies on either focused PCD newborn screening or, failing this, a general report on NBS. We found over fifty-five suitable publications and gathered the following data, upon availability: country/region; period of the study; number of newborns screened; free carnitine (C_0_) cut-off for screening; second tier screening if performed; the number of patients diagnosed and their subsequent incidence of PCD; the number of mothers with PCD identified by their infant’s NBS; and the number of false positive patients and positive predictive value (PPV%). Data are presented by region rather than chronologically to ease comprehension in Table 1.

From these data, we calculated regional incidences of 1:348,333 for Australia and New-Zealand (6:2,090,000 births); 1:121,609 for North America (306:37,212,366); 1:127,912 for Europe, excluding Denmark, Greenland, and Faroe Islands (59:7,546,812); and 1:50,386 for Asia (544:27,409,799). Incidences were compared to each other using a chi-square test of equal frequencies. Incidence was significantly higher in Asia compared to all other regions (*p* < 0.0001). Incidence in Europe did not differ from that in North America (*p* = 0.72), whereas Australia displayed a lower incidence compared with Europe (*p* = 0.015) and North America (*p* = 0.001). Among these studies, only a few countries used a secondary batch of biomarkers in order to reduce false positive rates. For example, Tang et al. and Huang et al. [97,105] showed that using the sum of propionylcarnitine and palmitoylcarnitine on DBS (C_3_ + C_16_) < 2 µmol·L^−1^ cut-off, in addition to a C_0_ cut-off of 10 µmol·L^−1^, allowed sensitivity which was as effective as using a sole C_0_ < 8.5 µmol·L^−1^ cut-off, while reducing false positives samples from 314 to 165. The positive predictive value (PPV%) increased from 8.7 to 15.3%. It is noteworthy that some countries, such as China, Norway, or Slovenia, have decided to use a next-generation sequencing research of common pathogenic variants on DBS as a second-tier screening. The main argument to this is the need to identify heterozygous newborns, and thus excluding false positive children born from PCD mothers. This leads to an increase in PPV%, which is generally low for PCD. However, the use of second tier biomarkers or molecular tests raise PPV% to almost 20%.

## 4. Molecular Findings

From the above-mentioned studies, we gathered individual molecular data that were available [58,64,72,73,78,80,83,86,89,92,96,98,101,103,104,107,110]. Genotype data were described for 175 newborns and C_0_ levels on screening were mentioned for 132 of the newborns. Seventy-five unique variants were identified: 49 missense, 10 nonsense, 10 frameshift, 10 intronic, and 1 in frame. The most prevalent variants were c.1400C>G, p.(Ser467Cys), representing 79 alleles (22.6%) out of 350, followed by c.760C>T p.(Arg254*), 56 alleles (16.0%); and c.51C>G p.(Phe17Leu), 49 alleles (14%). Unfortunately, most of the data came from Asian studies because of the limited molecular reports from other areas, which does not allow a global overview. In addition, databases on population allele frequency (GnomAD) confirmed that these variants were prevalent in East-Asian population, but not in other populations. Interestingly, in the study by Martín-Rivada et al., p.(Ser467Cys) and p.(Arg254*) were only present once out of 22 alleles and both in the same patient, who originated from China, whereas other described variants were not present in Asian studies, in accordance with a region-dependent polymorphism. Therefore, we cannot postulate on a global genotype-phenotype correlation between the pathogenic variants found in PCD patients who were identified through newborn screening. Nevertheless, it is interesting to note that in these data, C_0_ values for homozygous or composite heterozygous missense variants displayed milder decreased free carnitine levels (mean C_0_ ± Sd = 5.92 ± 1.76 µmol·L^−1^), which is consistent with the literature on its effect on residual OCTN2 activity [111]. On the contrary, truncating genotypes were more deleterious, with a mean C_0_ level below 3 µmol·L^−1^ at the homozygous state. Hence, in order to increase the positive predictive value and sensitivity of NBS, it is desirable to know the molecular distribution within a population to set an appropriate cut-off for the biomarker of interest.

## 5. Pitfalls of Newborn Screening for PCD

Including primary carnitine deficiency to NBS is not as simple as it is for other inborn errors of metabolism. Indeed, PCD is eligible based on the Wilson and Jungner criteria, as it is an easily treatable and very serious condition. Nonetheless, there are substantial obstacles. It is one of the rare diseases for which the screening biomarker is not expected to be detectable above a cut-off value, but below. This is a major problem as there are common causes of decreased free carnitine levels in a newborn, such as: preterm birth [50], maternal PCD, inborn errors of metabolism or vegetarian/vegan diet [112,113], and pivalic acid-based therapeutics in the mother (e.g., pivmecillinam, cephalosporin antibiotics, sivelestat, etc.) [114,115]. These situations are causes of false positive screening test results.

In addition, preanalytical issues can impact the DBS test. For example, the extraction method, using derivatization or not, will lead to different levels of C_0_, which are higher with derivatized methods [95]. Therefore, there is a need for standardization, at least for screening centers within the same country. The timing of blood collection is crucial as well, as C_0_ seems to decrease during the first 48 h of life before increasing until 120 h after [116,117]. Ethnicity can be a variating factor as well, as Asian populations seem to have higher C_0_ levels [118].

Particularly, the high incidence of identification of asymptomatic mothers with PCD from their child’s NBS, raises the question of the limit of the screening approach. Associated with the low sensitivity and positive predictive value of PCD NBS, it would lead to the diagnosis of more mothers than children. For this reason, and the possible side effects of a long term supplementation with L-carnitine, such as trimethyl-*N*-oxide (TMAO) accumulation and repression of compensation mechanisms observed in PCD, New-Zealand decided to discontinue PCD from their NBS program [25].

In summary, newborn screening for PCD is undoubtedly useful, but only if the following requirements are fulfilled: an algorithm to reduce false positive results, and to increase the positive predictive value by utilizing 1st, 2nd, and even 3rd tier analyses comprising C_0_ test and retest, along with 2nd tier biomarkers, and molecular and/or functional studies.

## 6. Discussion of a Suitable Screening Algorithm

The experience reported in the literature on PCD NBS confirmed its complexity. In order to propose a suitable algorithm for France, with an optimal PPV%, some lines of thought were identified such as:

- Using supplemental biomarkers in addition to C_0._
- Coupling *SLC22A5* molecular testing on initial DBS.
- Performing a retest on a second DBS sample to prevent the impact of the maternal status.

**Figure 2:**
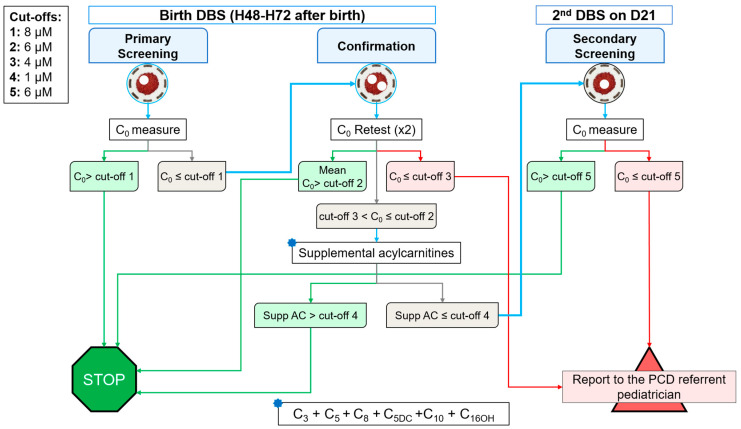
The three-step screening algorithm for PCD as proposed by the CNCDN and G2M working group. Cut-offs are set for the method using underivatized-based sample preparation.

A national working group proposed to use C_0_ as a first-tier biomarker, and total acylcarnitines as a second-step confirmation analysis (including acylcarnitines measured in other screened conditions: propionylcarnitine C_3_, isovalerylcarnitine C_5_, octanoylcarnitine C_8_, glutarylcarnitine C_5DC_, decanoyl C_10_, and 3-OH palmitoylcarnitine C_16OH_). A novel C_0_ analysis on a new DBS sample at day 21 of life was selected as a third-tier analysis to limit false positives due to maternal PCD, prematurity, or other inborn errors of metabolism. As illustrated in Figure 2:

- A C_0_ higher (>) than cut-off 1 (8 µmol·L^−1^) means a negative screening, and the patient is ruled-out.
- If C_0_ is lower or equal (≤) to cut-off 1, a duplicate retest on the birth DBS is performed.
- If mean C_0_ of this retest is >cut-off 2 (6 µmol·L^−1^), the patient is ruled-out.
- If mean C_0_ is ≤cut-off 3 (4 µmol·L^−1^), the screening is positive and the patient is reported to the PCD referent pediatrician.
- If C_0_ is between cut-offs 2 and 3, the sum of supplemental acylcarnitines is calculated.
- If supplemental acylcarnitines are >cut-off 4 (1 µmol·L^−1^), the patient is ruled-out.
- If supplemental acylcarnitines are ≤cut-off 4, another DBS is sampled at day 21 of life.
- If C_0_ on the 2nd DBS sample is >cut-off 5 (6 µmol·L^−1^), the patient is ruled-out.
- If C_0_ is ≤cut-off 5, the screening is positive and the patient is reported to the PCD referent pediatrician.

It is to be noted that our algorithm was designed for full-term babies. Pre-term babies are known to have lower C_0_ levels and there is a physiological decrease from birth until 48 h after birth [116]. Thus, there is a risk of false positives in this population. Ramaswamy et al. showed that secondary carnitine deficiency due to carnitine depletion in pre-term babies did not exceed 10%, which is reassuring [50]. In addition, the third step with a new sample at day 21 after birth, will help to rule out these newborns and avoid entering the diagnosis process. On the other hand, total parenteral nutrition (TPN) can be a confounding factor too, as it can lead to false negatives if the preparation is supplemented with carnitine. In France, TPN are not carnitine enriched, therefore, it is likely that the risk of false negatives is very low.

## 7. Conclusions

Primary carnitine deficiency is a complex inborn error of metabolism. Firstly, this is because it involves a membrane transporter, which is polyspecific of a large panel of substrates, as opposed to enzymatic deficiencies mainly concerning only a single substrate. This implies that in addition to the hereditary defect due to deleterious biallelic variants in *SLC22A5*, several secondary etiologies can jeopardize a screening approach. Moreover, this disease displays variable expressivity. Indeed, almost half of the patients with PCD remain asymptomatic. Nonetheless, PCD is a permanent threat with a risk of sudden death in the event of decompensation. Furthermore, there is an efficient, cost-effective, and safe treatment through L-carnitine supplementation. If started at the pre-symptomatic phase, it can even prevent any onset of PCD symptoms. Finally, there are sensitive and specific screening and diagnosis tools with appropriate algorithms. For these reasons, a nationwide newborn screening program for this disease seems legitimate. Through this literature review, we provide a worldwide overview on current practices of PCD newborn screening and emphasize the pitfalls of this particular disease. We also present the algorithm that working groups for French newborn screening program have deemed the most appropriate.

## Figures and Tables

**Table 1:** Retrospective studies on PCD screening experience.

Reference	Country/Region	Period/ Survey Duration	Newborns Screened	First Tier Test C_0_ Threshold (µmol·L^−1^)		Secondary Markers	Number of Patients Diagnosed (Incidence)	Number of Maternal PCD Identified	False Positive Tests (PPV%)
*Australia and New-Zealand*									
[18]	New South Wales (Australia)	1998–2000	1,490,000	<10 (<5 *)			4 (1:372,500)	ND	1017 (0.4%)
[24,25]	New Zealand	2006–2016	~600,000	<5/5			2 (1:300,000)	9	73 (2.7%)
*North America*									
[62]	North Carolina (USA)	1997–2005	944,078	<13			0 (<1:944,078)	ND	0
[63]	USA	2001–2011	20,908,664	ND			147 (1:142,236)		
[64]	California (USA)	2005–2012	3,608,768	<12 (derivatized)			48 screened (1:75,000) 21 confirmed (1:172,000)	6	1030 (4.7%)
				<7 (underivatized)					
[20]	USA	2015–2017	11,750,856	ND			138 (1:85,151)		
*Europe*									
[65]	Tuscany (Italy)	2002–2004	160,000	<8			1 (1:160,000)	1	1 (50%)
[42]	England	2.5 years	24,983	<2 (Cord blood)			0 (<1:24,983)	2	2
[38]	Germany	1998–2001	250,000	<10		Sum of (C_3_–C_18_) < 5 µmol·L^−1^	1 (1:250,000)	ND	86 (1.2%)
[66]	Portugal	4 years	316,243	<7			4 (1:79,060)	1	1
[67]	Danmark Faroe Islands Greenland	2002–2011	504,049	<5.7		C_5_ < 0.43 µmol·L^−1^ AC/Cit < 3.0	5 (1:100,809)	8	28 (15%)
[68]	Austria	2002–2009	622,489	ND			2 (1:311,245)	ND	ND
[69]	Greece	2007–2009	45,000	<6.25			0 (<1:45,000)	ND	ND
[70]	Germany	1999–2009	1,084,195	<10			3 (1:361,398)	ND	ND
[71]	Galicia (Spain)	2000–2015	210,165	<9.5			1 (1:210,165)	ND	ND
[72]	Slovenia	2013–2014	10,048	<7.7		AC/Cit < 1.9	0 (<1:10,048)	ND	ND
[73]	Norway	2012–2020	461,369	<6		C_3_ + C_16_ > 2 µmol·L^−1^	3 (1:153,790)	2	22 (12%)
[74]	Verona (Italy)	2014–2019	86,320	ND			3 (1:28,773)	ND	ND
[75]	Danmark Faroe Islands Greenland	2002–2018	967,780	C_0_ < 5.7		C_5_ < 0.43 µmol·L^−1^ Ac/Cit < 3.0	40 (1:24,195) 32 TP + 8 FN	19	114 (21.9%)
[76]	Sicilia	2011–2017	60,408	ND			0 (1:60,408)	ND	ND
[77]	Sweden	2011–2019	1,000,000	ND			13 (1:76,923)	6	94 (12%)
[78,79]	Madrid (Spain)	2011–2019	592,822	C_0_ < ND AC/Cit < ND			12 (1:49,402)	ND	73 (14%)
[80]	Bavaria (Germany)	1999–2018	1,816,000	<9			6 (1:302,667)	12	151 (3.8%)
[81]	Italy	2017–2020	806,770	ND		Total AC	10 (1:80,677)	20	ND
*Asia*									
[82]	Taiwan	2000–2009	592,717	<8 Or <5	(<2 *)	Recall DBS	5 confirmed (1:118,543) +2 unconfirmed	0	111 (4.3%)
[83]	Taiwan	2001–2005	304,536	<2.6 <2.86 <10.95 <6.44 <8		Recall DBS	4 (1:67,000) ^‡^	6	12 (25%)
		2006	88,200						
		2007/1–2007/5	31,329						
		2007/6–2007/12	59,785						
		2008/1–2009/7	110,962						
[84]	Taiwan	2003–2012	790,569	<6.44			22 (1:35,934)	12	ND
[58]	Taiwan	6 months	30,237	<12 (<6.0 *)			1 (1:30,237)	0	209 (0.48%)
[85]	Singapore	2006–2014	117,267	<8		C_2_ < 7 µmol·L^−1^	5 (1:35,453)	5	20 (20%)
[86]	Nanjing (China)	2013–2016	62,568	<10			7 (1:8,938)	ND	ND
[87]	Hong Kong	2013–2016	30,448	<6.4			0 (<1:30448)	1	17
[88]	Zhejiang (China)	2009–2016	1,861,262	ND			78 (1:23,350)	ND	ND
[55]	Thailand	2014–2017	99,234	ND			5 (1: 372,252)	6	ND
[89]	Jining (China)	2015	48,287	ND			5 (1:9,657)	ND	ND
[90]	Japan	1997–2015	3,360,000	ND			17 (1:199,000)	ND	ND
	Taiwan	2001–2014	1,390,000				20 (1:70,000)		
	South Korea	2000–2015	3,440,000				10 (1:345,000)		
	Germany	2002–2015	7,510,000				30 (1:250,000)		
[91]	Seoul (South Korea)	2002–2016	ND	<12			1 (ND)	ND	ND
[92]	Xuzhou (China)	2015–2017	236,368	<9.63 (<5 *)			10 (1:23,637)	6	176 (5.4%)
[93]	Suzhou (China)	2014–2018	401,660	<9.5			15 (1:26,777)	ND	ND
[94]	Beijing (China)	2014–2019	58,651	<10			1 (1:58,651)	ND	ND
[95]	Zhejiang (China)	2009–2019	3,410,600	<14 (derivatized)			113 (1:30,182)	63	ND
				<10.28 (underivatized)					
[96]	Tianjin (China)	2013–2018	220,443	<13 (derivatized)			10 (1:22,044)	ND	ND
[97]	Guangzhou (China)	2015–2019	200,180	<10		C_0_ < 8.5 µmol·L^−1^ Or C_0_ [8.5–10] And C_3_ + C_16_ < 2 µmol·L^−1^	15 (1:13,345)	22	239 (5.9%)
[98]	Fujian (China)	2015–2020	94,453	<8.8			9 (1:10,495)	1	ND
[60,99,100,101]	Quanzhou (China)	2014–2021	548,247	<8.5			49 (1:11,189)	6	1665 (2.9%)
[102]	Ningbo (China)	2014–2018	265,524	<9.5			16 (1:16,595)	3 confirmed + 7 unconfirmed	1669 (0.96%)
[103]	Liuzhou (China)	2012–2020	111,986	<9			12 (1:9,332)		2452 (0.49%)
[104]	Jining (China)	2014–2019	608,818	<10			16 (1:38,051)	ND	ND
[105]	Guangzhou (China)	2015–2020	272,117	<10		(1) C_0_ < 8.5 µmol·L^−1^ Or (2) C_0_ [8.5–10] And C_3_ + C_16_ < 2 µmol·L^−1^	21 (1:12,958)	30	(1) 314 (8.7%) (2) 165 (15.3%)
[106]	Guangxi (China)	2014–2018	400,575	ND			22 (1:18,208)	9	ND
[107]	Changsha (China)	2016–2020	300,849	<8.5			22 (13,675)	ND	ND
[108]	Shaanxi (China)	2014–2019	146,152	<8.5			3 (1:48,717)	2	ND
[56]	Philippines	2005–2011	111,127	ND			0 (<1:111,127)	ND	ND
[109]	Shaoyang (Chine)	2016–2020	94,648	ND			5 (1:18,930)	ND	474 (1%)

*: Second threshold at retest in case of positivity (i.e., action cut-off); PPV%: Positive predictive value (%); ND: Not determined; ‡: Calculated by authors based on changes in protocol; p/sAC: plasma/serum acylcarnitines profile; AC/Cit: Acylcarnitines/Citrulline ratio; TP: True positive; FN: False negative; uC_0_: urinary free carnitine.

## Data Availability

Not applicable.

## References

[1] Wu X., Prasad P.D., Leibach F.H., Ganapathy V. (1998). CDNA Sequence, Transport Function, and Genomic Organization of Human OCTN2, a New Member of the Organic Cation Transporter Family. Biochem. Biophys. Res. Commun..

[2] Nezu J., Tamai I., Oku A., Ohashi R., Yabuuchi H., Hashimoto N., Nikaido H., Sai Y., Koizumi A., Shoji Y. (1999). Primary Systemic Carnitine Deficiency Is Caused by Mutations in a Gene Encoding Sodium Ion-Dependent Carnitine Transporter. Nat. Genet..

[3] Juraszek B., Nałęcz K.A. (2020). SLC22A5 (OCTN2) Carnitine Transporter—Indispensable for Cell Metabolism, a Jekyll and Hyde of Human Cancer. Molecules.

[4] Tamai I., Ohashi R., Nezu J., Yabuuchi H., Oku A., Shimane M., Sai Y., Tsuji A. (1998). Molecular and Functional Identification of Sodium Ion-Dependent, High Affinity Human Carnitine Transporter OCTN2*. J. Biol. Chem..

[5] Stanley C.A. (2004). Carnitine Deficiency Disorders in Children. Ann. N. Y. Acad. Sci..

[6] Longo N. (2016). Primary Carnitine Deficiency and Newborn Screening for Disorders of the Carnitine Cycle. Ann. Nutr. Metab..

[7] Longo N., Frigeni M., Pasquali M. (2016). Carnitine Transport and Fatty Acid Oxidation. Biochim. Biophys. Acta BBA-Mol. Cell Res..

[8] Stanley C.A., DeLeeuw S., Coates P.M., Vianey-Liaud C., Divry P., Bonnefont J.P., Saudubray J.M., Haymond M., Trefz F.K., Breningstall G.N. (1991). Chronic Cardiomyopathy and Weakness or Acute Coma in Children with a Defect in Carnitine Uptake. Ann. Neurol..

[9] El-Hattab A.W., Scaglia F. (2015). Disorders of Carnitine Biosynthesis and Transport. Mol. Genet. Metab..

[10] El-Hattab A.W., Adam M.P., Mirzaa G.M., Pagon R.A., Wallace S.E., Bean L.J., Gripp K.W., Amemiya A. (1993). Systemic Primary Carnitine Deficiency. GeneReviews^®^.

[11] Crefcoeur L.L., Visser G., Ferdinandusse S., Wijburg F.A., Langeveld M., Sjouke B. (2022). Clinical Characteristics of Primary Carnitine Deficiency—A Structured Review Using a Case by Case Approach. J. Inherit. Metab. Dis..

[12] Rasmussen J., Nielsen O.W., Janzen N., Duno M., Køber L., Steuerwald U., Lund A.M. (2014). Carnitine Levels in 26,462 Individuals from the Nationwide Screening Program for Primary Carnitine Deficiency in the Faroe Islands. J. Inherit. Metab. Dis..

[13] Rose E.C., di San Filippo C.A., Ndukwe Erlingsson U.C., Ardon O., Pasquali M., Longo N. (2012). Genotype-Phenotype Correlation in Primary Carnitine Deficiency. Hum. Mutat..

[14] Koizumi A., Nozaki J., Ohura T., Kayo T., Wada Y., Nezu J., Ohashi R., Tamai I., Shoji Y., Takada G. (1999). Genetic Epidemiology of the Carnitine Transporter OCTN2 Gene in a Japanese Population and Phenotypic Characterization in Japanese Pedigrees with Primary Systemic Carnitine Deficiency. Hum. Mol. Genet..

[15] Wilcken B., ChB M. (2008). Disorders of the Carnitine Cycle and Detection by Newborn Screening. Ann. Acad. Med. Singap..

[16] Dobrow M.J., Hagens V., Chafe R., Sullivan T., Rabeneck L. (2018). Consolidated Principles for Screening Based on a Systematic Review and Consensus Process. CMAJ Can. Med. Assoc. J..

[17] Barns R.J., Bowling F.G., Brown G., Clague A.E., Thompson A. (1991). Carnitine in Dried Blood Spots: A Method Suitable for Neonatal Screening. Clin. Chim. Acta.

[18] Wilcken B., Wiley V., Sim K.G., Carpenter K. (2001). Carnitine Transporter Defect Diagnosed by Newborn Screening with Electrospray Tandem Mass Spectrometry. J. Pediatr..

[19] Bodamer O.A., Hoffmann G.F., Lindner M. (2007). Expanded Newborn Screening in Europe 2007. J. Inherit. Metab. Dis..

[20] Sontag M.K., Yusuf C., Grosse S.D., Edelman S., Miller J.I., McKasson S., Kellar-Guenther Y., Gaffney M., Hinton C.F., Cuthbert C. (2020). Infants with Congenital Disorders Identified Through Newborn Screening—United States, 2015–2017. MMWR Morb. Mortal. Wkly. Rep..

[21] Koracin V., Mlinaric M., Baric I., Brincat I., Djordjevic M., Drole Torkar A., Fumic K., Kocova M., Milenkovic T., Moldovanu F. (2021). Current Status of Newborn Screening in Southeastern Europe. Front. Pediatr..

[22] Haute Autorité de Santé: Évaluation a priori de l’extension du dépistage néonatal à une ou plusieurs erreurs innées du métabolisme par spectrométrie de masse en tandem en population générale en France. https://www.has-sante.fr/jcms/c_2866458/fr/evaluation-a-priori-de-l-extension-du-depistage-neonatal-a-une-ou-plusieurs-erreurs-innees-du-metabolisme-par-spectrometrie-de-masse-en-tandem-volet-2.

[23] Care A.G.D. List of Conditions Screened by Jurisdiction—September 2022. https://www.health.gov.au/resources/publications/list-of-conditions-screened-by-jurisdiction-september-2022?language=en.

[24] Wilson C., Kerruish N.J., Wilcken B., Wiltshire E., Webster D. (2012). Diagnosis of Disorders of Intermediary Metabolism in New Zealand before and after Expanded Newborn Screening: 2004–2009. N. Z. Med. J..

[25] Wilson C., Knoll D., de Hora M., Kyle C., Glamuzina E., Webster D. (2019). The Decision to Discontinue Screening for Carnitine Uptake Disorder in New Zealand. J. Inherit. Metab. Dis..

[26] Therrell B.L., Hannon W.H. (2006). National Evaluation of US Newborn Screening System Components. Ment. Retard. Dev. Disabil. Res. Rev..

[27] Watson M.S., Mann M.Y., Lloyd-Puryear M.A., Rinaldo P., Howell R.R. (2006). American College of Medical Genetics Newborn Screening Expert Group Newborn Screening: Toward a Uniform Screening Panel and System—Executive Summary. Pediatrics.

[28] (2008). Centers for Disease Control and Prevention (CDC) Impact of Expanded Newborn Screening--United States, 2006. MMWR Morb. Mortal. Wkly. Rep..

[29] (2012). Centers for Disease Control and Prevention (CDC) CDC Grand Rounds: Newborn Screening and Improved Outcomes. MMWR Morb. Mortal. Wkly. Rep..

[30] Magoulas P.L., El-Hattab A.W. (2012). Systemic Primary Carnitine Deficiency: An Overview of Clinical Manifestations, Diagnosis, and Management. Orphanet J. Rare Dis..

[31] Ojodu J., Singh S., Kellar-Guenther Y., Yusuf C., Jones E., Wood T., Baker M., Sontag M. (2017). NewSTEPs: The Establishment of a National Newborn Screening Technical Assistance Resource Center. Int. J. Neonatal Screen..

[32] Hanley W.B. (2005). Newborn Screening in Canada—Are We out of Step?. Paediatr. Child Health.

[33] Dyack S. (2004). Expanded Newborn Screening: Lessons Learned from MCAD Deficiency. Paediatr. Child Health.

[34] Auray-Blais C., Boutin M., Lavoie P., Maranda B. (2021). Neonatal Urine Screening Program in the Province of Quebec: Technological Upgrade from Thin Layer Chromatography to Tandem Mass Spectrometry. Int. J. Neonatal Screen..

[35] Borrajo G.J.C. (2007). Newborn Screening in Latin America at the Beginning of the 21st Century. J. Inherit. Metab. Dis..

[36] Cabello J.F., Novoa F., Huff H.V., Colombo M. (2021). Expanded Newborn Screening and Genomic Sequencing in Latin America and the Resulting Social Justice and Ethical Considerations. Int. J. Neonatal Screen..

[37] Borrajo G.J.C. (2021). Newborn Screening in Latin America: A Brief Overview of the State of the Art. Am. J. Med. Genet. C Semin. Med. Genet..

[38] Schulze A., Lindner M., Kohlmüller D., Olgemöller K., Mayatepek E., Hoffmann G.F. (2003). Expanded Newborn Screening for Inborn Errors of Metabolism by Electrospray Ionization-Tandem Mass Spectrometry: Results, Outcome, and Implications. Pediatrics.

[39] Lund A.M., Joensen F., Hougaard D.M., Jensen L.K., Christensen E., Christensen M., Nørgaard-Petersen B., Schwartz M., Skovby F. (2007). Carnitine Transporter and Holocarboxylase Synthetase Deficiencies in The Faroe Islands. J. Inherit. Metab. Dis..

[40] Steuerwald U., Lund A.M., Rasmussen J., Janzen N., Hougaard D.M., Longo N. (2017). Neonatal Screening for Primary Carnitine Deficiency: Lessons Learned from the Faroe Islands. Int. J. Neonatal Screen..

[41] Loeber J.G., Platis D., Zetterström R.H., Almashanu S., Boemer F., Bonham J.R., Borde P., Brincat I., Cheillan D., Dekkers E. (2021). Neonatal Screening in Europe Revisited: An ISNS Perspective on the Current State and Developments Since 2010. Int. J. Neonatal Screen..

[42] Walter J.H., Patterson A., Till J., Besley G.T.N., Fleming G., Henderson M.J. (2009). Bloodspot Acylcarnitine and Amino Acid Analysis in Cord Blood Samples: Efficacy and Reference Data from a Large Cohort Study. J. Inherit. Metab. Dis..

[43] Jansen M.E., Klein A.W., Buitenhuis E.C., Rodenburg W., Cornel M.C. (2021). Expanded Neonatal Bloodspot Screening Programmes: An Evaluation Framework to Discuss New Conditions With Stakeholders. Front. Pediatr..

[44] Therrell B.L., Padilla C.D., Loeber J.G., Kneisser I., Saadallah A., Borrajo G.J.C., Adams J. (2015). Current Status of Newborn Screening Worldwide: 2015. Semin. Perinatol..

[45] Al Hosani H., Salah M., Osman H.M., Farag H.M., El Assiouty L., Saade D., Hertecant J. (2014). Expanding the Comprehensive National Neonatal Screening Programme in the United Arab Emirates from 1995 to 2011. East. Mediterr. Health J..

[46] Golbahar J., Al-Jishi E.A., Altayab D.D., Carreon E., Bakhiet M., Alkhayyat H. (2013). Selective Newborn Screening of Inborn Errors of Amino Acids, Organic Acids and Fatty Acids Metabolism in the Kingdom of Bahrain. Mol. Genet. Metab..

[47] Hassan F.A., El-Mougy F., Sharaf S.A., Mandour I., Morgan M.F., Selim L.A., Hassan S.A., Salem F., Oraby A., Girgis M.Y. (2016). Inborn Errors of Metabolism Detectable by Tandem Mass Spectrometry in Egypt: The First Newborn Screening Pilot Study. J. Med. Screen..

[48] Mohamed S., Elsheikh W., Al-Aqeel A.I., Alhashem A.M., Alodaib A., Alahaideb L., Almashary M., Alharbi F., AlMalawi H., Ammari A. (2020). Incidence of Newborn Screening Disorders among 56632 Infants in Central Saudi Arabia: A 6-Year Study. Saudi Med. J..

[49] Lindner M., Abdoh G., Fang-Hoffmann J., Shabeck N., Al Sayrafi M., Al Janahi M., Ho S., Abdelrahman M.O., Ben-Omran T., Bener A. (2007). Implementation of Extended Neonatal Screening and a Metabolic Unit in the State of Qatar: Developing and Optimizing Strategies in Cooperation with the Neonatal Screening Center in Heidelberg. J. Inherit. Metab. Dis..

[50] Ramaswamy M., Anthony Skrinska V., Fayez Mitri R., Abdoh G. (2019). Diagnosis of Carnitine Deficiency in Extremely Preterm Neonates Related to Parenteral Nutrition: Two Step Newborn Screening Approach. Int. J. Neonatal Screen..

[51] Therrell B.L., Lloyd-Puryear M.A., Ohene-Frempong K., Ware R.E., Padilla C.D., Ambrose E.E., Barkat A., Ghazal H., Kiyaga C., Mvalo T. (2020). Empowering Newborn Screening Programs in African Countries through Establishment of an International Collaborative Effort. J. Community Genet..

[52] Lamhonwah A.M., Tein I. (1998). Carnitine Uptake Defect: Frameshift Mutations in the Human Plasmalemmal Carnitine Transporter Gene. Biochem. Biophys. Res. Commun..

[53] Tang N.L.S., Hwu W.L., Chan R.T., Law L.K., Fung L.M., Zhang W.M. (2002). A Founder Mutation (R254X) of SLC22A5 (OCTN2) in Chinese Primary Carnitine Deficiency Patients. Hum. Mutat..

[54] Tang N., Hui J. (2020). 20 Years After Discovery of the Causative Gene of Primary Carnitine Deficiency, How Much More Have We Known About the Disease?. HK J Paediatr New Ser..

[55] Liammongkolkul S., Sanomcham K., Vatanavicharn N., Sathienkijkanchai A., Ranieri E., Wasant P. (2017). AB133. Expanded Newborn Screening Program in Thailand. Ann. Transl. Med..

[56] Padilla C.D., Therrell B.L., Alcausin M.M.L.B., Chiong M.A.D., Abacan M.A.R., Reyes M.E.L., Jomento C.M., Dizon-Escoreal M.T.T., Canlas M.A.E., Abadingo M.E. (2022). Successful Implementation of Expanded Newborn Screening in the Philippines Using Tandem Mass Spectrometry. Int. J. Neonatal Screen..

[57] Mookken T. (2020). Universal Implementation of Newborn Screening in India. Int. J. Neonatal Screen..

[58] Wang L.-Y., Chen N.-I., Chen P.-W., Chiang S.-C., Hwu W.-L., Lee N.-C., Chien Y.-H. (2013). Newborn Screening for Citrin Deficiency and Carnitine Uptake Defect Using Second-Tier Molecular Tests. BMC Med. Genet..

[59] Huang X., Wu D., Zhu L., Wang W., Yang R., Yang J., He Q., Zhu B., You Y., Xiao R. (2022). Application of a Next-Generation Sequencing (NGS) Panel in Newborn Screening Efficiently Identifies Inborn Disorders of Neonates. Orphanet J. Rare Dis..

[60] Lin Y., Zhang W., Huang C., Lin C., Lin W., Peng W., Fu Q., Chen D. (2021). Increased Detection of Primary Carnitine Deficiency through Second-Tier Newborn Genetic Screening. Orphanet J. Rare Dis..

[61] Volgina S.Y., Sokolov A.A. (2021). An Analysis of Medical Care Services for Children With Rare Diseases in the Russian Federation. Front. Pharmacol..

[62] Frazier D.M., Millington D.S., McCandless S.E., Koeberl D.D., Weavil S.D., Chaing S.H., Muenzer J. (2006). The Tandem Mass Spectrometry Newborn Screening Experience in North Carolina: 1997–2005. J. Inherit. Metab. Dis..

[63] Therrell B.L., Lloyd-Puryear M.A., Camp K.M., Mann M.Y. (2014). Inborn Errors of Metabolism Identified via Newborn Screening: Ten-Year Incidence Data and Costs of Nutritional Interventions for Research Agenda Planning. Mol. Genet. Metab..

[64] Gallant N.M., Leydiker K., Wilnai Y., Lee C., Lorey F., Feuchtbaum L., Tang H., Carter J., Enns G.M., Packman S. (2017). Biochemical Characteristics of Newborns with Carnitine Transporter Defect Identified by Newborn Screening in California. Mol. Genet. Metab..

[65] La Marca G., Malvagia S., Casetta B., Pasquini E., Donati M.A., Zammarchi E. (2008). Progress in Expanded Newborn Screening for Metabolic Conditions by LC-MS/MS in Tuscany: Update on Methods to Reduce False Tests. J. Inherit. Metab. Dis..

[66] Vilarinho L., Rocha H., Sousa C., Marcão A., Fonseca H., Bogas M., Osório R.V. (2010). Four Years of Expanded Newborn Screening in Portugal with Tandem Mass Spectrometry. J. Inherit. Metab. Dis..

[67] Lund A.M., Hougaard D.M., Simonsen H., Andresen B.S., Christensen M., Dunø M., Skogstrand K., Olsen R.K.J., Jensen U.G., Cohen A. (2012). Biochemical Screening of 504,049 Newborns in Denmark, the Faroe Islands and Greenland—Experience and Development of a Routine Program for Expanded Newborn Screening. Mol. Genet. Metab..

[68] Kasper D.C., Ratschmann R., Metz T.F., Mechtler T.P., Möslinger D., Konstantopoulou V., Item C.B., Pollak A., Herkner K.R. (2010). The National Austrian Newborn Screening Program–Eight Years Experience with Mass Spectrometry. Past, Present, and Future Goals. Wien. Klin. Wochenschr..

[69] Loukas Y.L., Soumelas G.-S., Dotsikas Y., Georgiou V., Molou E., Thodi G., Boutsini M., Biti S., Papadopoulos K. (2010). Expanded Newborn Screening in Greece: 30 Months of Experience. J. Inherit. Metab. Dis..

[70] Lindner M., Gramer G., Haege G., Fang-Hoffmann J., Schwab K.O., Tacke U., Trefz F.K., Mengel E., Wendel U., Leichsenring M. (2011). Efficacy and Outcome of Expanded Newborn Screening for Metabolic Diseases-Report of 10 Years from South-West Germany *. Orphanet J. Rare Dis..

[71] Couce M.L., Castiñeiras D.E., Bóveda M.D., Baña A., Cocho J.A., Iglesias A.J., Colón C., Alonso-Fernández J.R., Fraga J.M. (2011). Evaluation and Long-Term Follow-up of Infants with Inborn Errors of Metabolism Identified in an Expanded Screening Programme. Mol. Genet. Metab..

[72] Smon A., Repic Lampret B., Groselj U., Zerjav Tansek M., Kovac J., Perko D., Bertok S., Battelino T., Trebusak Podkrajsek K. (2018). Next Generation Sequencing as a Follow-up Test in an Expanded Newborn Screening Programme. Clin. Biochem..

[73] Tangeraas T., Sæves I., Klingenberg C., Jørgensen J., Kristensen E., Gunnarsdottir G., Hansen E.V., Strand J., Lundman E., Ferdinandusse S. (2020). Performance of Expanded Newborn Screening in Norway Supported by Post-Analytical Bioinformatics Tools and Rapid Second-Tier DNA Analyses. Int. J. Neonatal Screen..

[74] Maguolo A., Rodella G., Dianin A., Nurti R., Monge I., Rigotti E., Cantalupo G., Salviati L., Tucci S., Pellegrini F. (2020). Diagnosis, Genetic Characterization and Clinical Follow up of Mitochondrial Fatty Acid Oxidation Disorders in the New Era of Expanded Newborn Screening: A Single Centre Experience. Mol. Genet. Metab. Rep..

[75] Lund A., Wibrand F., Skogstrand K., Cohen A., Christensen M., Jäpelt R.B., Dunø M., Skovby F., Nørgaard-Pedersen B., Gregersen N. (2020). Danish Expanded Newborn Screening Is a Successful Preventive Public Health Programme. Dan. Med. J..

[76] Messina M., Meli C., Raudino F., Pittalá A., Arena A., Barone R., Giuffrida F., Iacobacci R., Muccilli V., Sorge G. (2018). Expanded Newborn Screening Using Tandem Mass Spectrometry: Seven Years of Experience in Eastern Sicily. Int. J. Neonatal Screen..

[77] Sörensen L., von Döbeln U., Åhlman H., Ohlsson A., Engvall M., Naess K., Backman-Johansson C., Nordqvist Y., Wedell A., Zetterström R.H. (2020). Expanded Screening of One Million Swedish Babies with R4S and CLIR for Post-Analytical Evaluation of Data. Int. J. Neonatal Screen..

[78] Martín-Rivada Á., Palomino Pérez L., Ruiz-Sala P., Navarrete R., Cambra Conejero A., Quijada Fraile P., Moráis López A., Belanger-Quintana A., Martín-Hernández E., Bellusci M. (2022). Diagnosis of Inborn Errors of Metabolism within the Expanded Newborn Screening in the Madrid Region. JIMD Rep..

[79] Conejero A.C., Figueras L.M., Temprado A.O., Soto P.B., Martín Á., Pérez L.P., Villarroya E.C., Giner C.P., Fraile P.Q., Martín-Hernández E. (2020). Análisis de Casos positivos de cribado neonatal de errores congénitos del metabolismo en la comunidad de madrid. Rev. Esp. Salud. Pública.

[80] Schiergens K.A., Weiss K.J., Röschinger W., Lotz-Havla A.S., Schmitt J., Dalla Pozza R., Ulrich S., Odenwald B., Kreuder J., Maier E.M. (2021). Newborn Screening for Carnitine Transporter Defect in Bavaria and the Long-Term Follow-up of the Identified Newborns and Mothers: Assessing the Benefit and Possible Harm Based on 19 ½ Years of Experience. Mol. Genet. Metab. Rep..

[81] Ruoppolo M., Malvagia S., Boenzi S., Carducci C., Dionisi-Vici C., Teofoli F., Burlina A., Angeloni A., Aronica T., Bordugo A. (2022). Expanded Newborn Screening in Italy Using Tandem Mass Spectrometry: Two Years of National Experience. Int. J. Neonatal Screen..

[82] Niu D.-M., Chien Y.-H., Chiang C.-C., Ho H.-C., Hwu W.-L., Kao S.-M., Chiang S.-H., Kao C.-H., Liu T.-T., Chiang H. (2010). Nationwide Survey of Extended Newborn Screening by Tandem Mass Spectrometry in Taiwan. J. Inherit. Metab. Dis..

[83] Lee N.-C., Tang N.L.-S., Chien Y.-H., Chen C.-A., Lin S.-J., Chiu P.-C., Huang A.-C., Hwu W.-L. (2010). Diagnoses of Newborns and Mothers with Carnitine Uptake Defects through Newborn Screening. Mol. Genet. Metab..

[84] Chien Y.-H., Lee N.-C., Chao M.-C., Chen L.-C., Chen L.-H., Chien C.-C., Ho H.-C., Suen J.-H., Hwu W.-L., Zschocke J., Gibson K.M., Brown G., Morava E., Peters V. (2013). Fatty Acid Oxidation Disorders in a Chinese Population in Taiwan. JIMD Reports-Volume 11.

[85] Lim J.S., Tan E.S., John C.M., Poh S., Yeo S.J., Ang J.S.M., Adakalaisamy P., Rozalli R.A., Hart C., Tan E.T.H. (2014). Inborn Error of Metabolism (IEM) Screening in Singapore by Electrospray Ionization-Tandem Mass Spectrometry (ESI/MS/MS): An 8year Journey from Pilot to Current Program. Mol. Genet. Metab..

[86] Sun Y., Wang Y.-Y., Jiang T. (2017). Clinical Features and Genotyping of Patients with Primary Carnitine Deficiency Identified by Newborn Screening. J. Pediatr. Endocrinol. Metab. JPEM.

[87] Chong S.C., Law L.K., Hui J., Lai C.Y., Leung T.Y., Yuen Y.P. (2017). Expanded Newborn Metabolic Screening Programme in Hong Kong: A Three-Year Journey. Hong Kong Med. J..

[88] Zheng J., Zhang Y., Hong F., Yang J., Tong F., Mao H., Huang X., Zhou X., Yang R., Zhao Z. (2017). Screening for fatty acid oxidation disorders of newborns in Zhejiang province:prevalence, outcome and follow-up. Zhejiang Xue Xue Bao Yi Xue Ban.

[89] Guo K., Zhou X., Chen X., Wu Y., Liu C., Kong Q. (2018). Expanded Newborn Screening for Inborn Errors of Metabolism and Genetic Characteristics in a Chinese Population. Front. Genet..

[90] Shibata N., Hasegawa Y., Yamada K., Kobayashi H., Purevsuren J., Yang Y., Dung V.C., Khanh N.N., Verma I.C., Bijarnia-Mahay S. (2018). Diversity in the Incidence and Spectrum of Organic Acidemias, Fatty Acid Oxidation Disorders, and Amino Acid Disorders in Asian Countries: Selective Screening vs. Expanded Newborn Screening. Mol. Genet. Metab. Rep..

[91] Kang E., Kim Y.-M., Kang M., Heo S.-H., Kim G.-H., Choi I.-H., Choi J.-H., Yoo H.-W., Lee B.H. (2018). Clinical and Genetic Characteristics of Patients with Fatty Acid Oxidation Disorders Identified by Newborn Screening. BMC Pediatr..

[92] Zhou W., Li H., Huang T., Zhang Y., Wang C., Gu M. (2019). Biochemical, Molecular, and Clinical Characterization of Patients With Primary Carnitine Deficiency via Large-Scale Newborn Screening in Xuzhou Area. Front. Pediatr..

[93] Wang T., Ma J., Zhang Q., Gao A., Wang Q., Li H., Xiang J., Wang B. (2019). Expanded Newborn Screening for Inborn Errors of Metabolism by Tandem Mass Spectrometry in Suzhou, China: Disease Spectrum, Prevalence, Genetic Characteristics in a Chinese Population. Front. Genet..

[94] Yang N., Gong L.-F., Zhao J.-Q., Yang H.-H., Ma Z.-J., Liu W., Wan Z.-H., Kong Y.-Y. (2020). Inborn Errors of Metabolism Detectable by Tandem Mass Spectrometry in Beijing. J. Pediatr. Endocrinol. Metab. JPEM.

[95] Lin Y., Xu H., Zhou D., Hu Z., Zhang C., Hu L., Zhang Y., Zhu L., Lu B., Zhang T. (2020). Screening 3.4 Million Newborns for Primary Carnitine Deficiency in Zhejiang Province, China. Clin. Chim. Acta Int. J. Clin. Chem..

[96] Wang S., Leng J., Diao C., Wang Y., Zheng R. (2020). Genetic Characteristics and Follow-up of Patients with Fatty Acid β-Oxidation Disorders through Expanded Newborn Screening in a Northern Chinese Population. J. Pediatr. Endocrinol. Metab. JPEM.

[97] Huang Y.L., Tang C.F., Liu S.C., Sheng H.Y., Tang F., Jiang X., Zheng R.D., Mei H.F., Liu L. (2020). Newborn screening for primary carnitine deficiency and variant spectrum of SLC22A5 gene in Guangzhou. Zhonghua Er Ke Za Zhi.

[98] Chen Y., Lin Q., Zeng Y., Qiu X., Liu G., Zhu W. (2021). Gene Spectrum and Clinical Traits of 10 Patients with Primary Carnitine Deficiency. Mol. Genet. Genomic Med..

[99] Lin Y., Zheng Q., Zheng T., Zheng Z., Lin W., Fu Q. (2019). Expanded Newborn Screening for Inherited Metabolic Disorders and Genetic Characteristics in a Southern Chinese Population. Clin. Chim. Acta Int. J. Clin. Chem..

[100] Lin W., Wang K., Zheng Z., Chen Y., Fu C., Lin Y., Chen D. (2021). Newborn Screening for Primary Carnitine Deficiency in Quanzhou, China. Clin. Chim. Acta Int. J. Clin. Chem..

[101] Lin Y., Lin B., Chen Y., Zheng Z., Fu Q., Lin W., Zhang W. (2021). Biochemical and Genetic Characteristics of Patients with Primary Carnitine Deficiency Identified through Newborn Screening. Orphanet J. Rare Dis..

[102] Yang X., Li Q., Wang F., Yan L., Zhuang D., Qiu H., Li H., Chen L. (2021). Newborn Screening and Genetic Analysis Identify Six Novel Genetic Variants for Primary Carnitine Deficiency in Ningbo Area, China. Front. Genet..

[103] Tan J., Chen D., Chang R., Pan L., Yang J., Yuan D., Huang L., Yan T., Ning H., Wei J. (2021). Tandem Mass Spectrometry Screening for Inborn Errors of Metabolism in Newborns and High-Risk Infants in Southern China: Disease Spectrum and Genetic Characteristics in a Chinese Population. Front. Genet..

[104] Yang C., Shi C., Zhou C., Wan Q., Zhou Y., Chen X., Jin X., Huang C., Xu P. (2021). Screening and Follow-up Results of Fatty Acid Oxidative Metabolism Disorders in 608 818 Newborns in Jining, Shandong Province. Zhejiang Xue Xue Bao Yi Xue Ban.

[105] Tang C., Tan M., Xie T., Tang F., Liu S., Wei Q., Liu J., Huang Y. (2021). Screening for Neonatal Inherited Metabolic Disorders by Tandem Mass Spectrometry in Guangzhou. Zhejiang Xue Xue Bao Yi Xue Ban.

[106] Geng G., Yang Q., Fan X., Lin C., Wu L., Chen S., Luo J. (2021). Analysis of metabolic profile and genetic variants for newborns with primary carnitine deficiency from Guangxi. Zhonghua Yi Xue Yi Chuan Xue Za Zhi.

[107] Li X., He J., He L., Zeng Y., Huang X., Luo Y., Li Y. (2021). Spectrum Analysis of Inherited Metabolic Disorders for Expanded Newborn Screening in a Central Chinese Population. Front. Genet..

[108] Zhang R., Qiang R., Song C., Ma X., Zhang Y., Li F., Wang R., Yu W., Feng M., Yang L. (2021). Spectrum Analysis of Inborn Errors of Metabolism for Expanded Newborn Screening in a Northwestern Chinese Population. Sci. Rep..

[109] Zhou M., Deng L., Huang Y., Xiao Y., Wen J., Liu N., Zeng Y., Zhang H. (2022). Application of the Artificial Intelligence Algorithm Model for Screening of Inborn Errors of Metabolism. Front. Pediatr..

[110] Luo X., Sun Y., Xu F., Guo J., Li L., Lin Z., Ye J., Gu X., Yu Y. (2020). A Pilot Study of Expanded Newborn Screening for 573 Genes Related to Severe Inherited Disorders in China: Results from 1,127 Newborns. Ann. Transl. Med..

[111] Frigeni M., Balakrishnan B., Yin X., Calderon F.R.O., Mao R., Pasquali M., Longo N. (2017). Functional and Molecular Studies in Primary Carnitine Deficiency. Hum. Mutat..

[112] El-Hattab A.W., Li F.-Y., Shen J., Powell B.R., Bawle E.V., Adams D.J., Wahl E., Kobori J.A., Graham B., Scaglia F. (2010). Maternal Systemic Primary Carnitine Deficiency Uncovered by Newborn Screening: Clinical, Biochemical, and Molecular Aspects. Genet. Med..

[113] Lin H.J., Neidich J.A., Salazar D., Thomas-Johnson E., Ferreira B.F., Kwong A.M., Lin A.M., Jonas A.J., Levine S., Lorey F. (2009). Asymptomatic Maternal Combined Homocystinuria and Methylmalonic Aciduria (CblC) Detected through Low Carnitine Levels on Newborn Screening. J. Pediatr..

[114] Holme E., Jodal U., Linstedt S., Nordin I. (1992). Effects of Pivalic Acid-Containing Prodrugs on Carnitine Homeostasis and on Response to Fasting in Children. Scand. J. Clin. Lab. Investig..

[115] Yamada K., Kobayashi H., Bo R., Takahashi T., Hasegawa Y., Nakamura M., Ishige N., Yamaguchi S. (2015). Elevation of Pivaloylcarnitine by Sivelestat Sodium in Two Children. Mol. Genet. Metab..

[116] Peng G., Tang Y., Cowan T.M., Zhao H., Scharfe C. (2020). Timing of Newborn Blood Collection Alters Metabolic Disease Screening Performance. Front. Pediatr..

[117] He F., Yang R., Huang X., Tian Y., Pei X., Bohn M.K., Zou L., Wang Y., Li H., Wang T. (2021). Reference Standards for Newborn Screening of Metabolic Disorders by Tandem Mass Spectrometry: A Nationwide Study on Millions of Chinese Neonatal Populations. Front. Mol. Biosci..

[118] Peng G., Tang Y., Gandotra N., Enns G.M., Cowan T.M., Zhao H., Scharfe C. (2020). Ethnic Variability in Newborn Metabolic Screening Markers Associated with False-positive Outcomes. J. Inherit. Metab. Dis..

